# Supplementary material for: Intravital deep-tumor single-beam 3-photon, 4-photon, and harmonic microscopy
Source: eLife. 2022 Feb 15;11:e63776. doi: 10.7554/eLife.63776 (PMC8849342; doi:10.7554/eLife.63776)
Supplement: Supplementary file 1. — (a) Comparison of parameters related to linear, lowIR, and highIR excitation modalities.The listed parameters are derived from experiments described in this paper, unless stated otherwise. Peak power focus is defined as the maximum excitation power in the focus during the laser pulse. Power surface is defined as the average excitation power at the sample surface calculated from the power under the objective and the water absorption (Figure 1—figure supplement 2). Emission I(E): dependence of emission intensity on excitation energy. Attenuation of light, confocal: very high, both excitation and detected emission (only ballistic photons from the focus pass the pinhole in the emission path) are strongly attenuated by the relatively short effective attenuation length for visible-range excitation (Helmchen and Denk, 2005; Benninger and Piston, 2013). LowIR: less attenuation as compared to visible excitation wavelengths; non-de-scanned detection improves emission detection (Centonze and White, 1998). HighIR: equal to less attenuation as compared to lowIR excitation. Water absorption: contribution of water absorption to the attenuation of excitation light (Qiu et al., 2017; Nachabé et al., 2010; Wang et al., 2016). (b) Order of the excitation processes (n) from emission intensity as a function of excitation energy (related to Figure 1). Data were derived from datasets described in Figure 1 and Figure 1—figure supplement 4. Additional independent measurements obtained by live imaging of multicellular HT1080 spheroids in rattail collagen included 1300 nm highIR excitation of eGFP, TagRFP, SHG, and THG and 1650 nm highIR excitation of TagRFP and mCherry at 0.5 and 1 MHz repetition rates. For fitting of data with 1300 nm excitation, the threshold for physical damage was 16 nJ and the saturation limit was 3.1 nJ for eGFP and TagRFP. Source data files: Figure 1—source data 1, Figure 1—source data 2, Source data 1 and Source data 2. (c) Experimental parameters for brain measureme [file elife-63776-supp1.docx]

Supplementary File 1

**Intravital Deep-Tumor Single-Beam 3-Photon, 4-Photon and Harmonic Microscopy**

Gert-Jan Bakker^1^, Sarah Weischer^1^, Júlia Ferrer Ortas^2^, Judith Heidelin^3^, Volker Andresen^3^, Marcus Beutler^4^, Emmanuel Beaurepaire^2^ and Peter Friedl^1,5,6^

^1^ Department of Cell Biology, Radboud Institute for Molecular Life Sciences, Radboud University Medical Centre, 6525 GA Nijmegen, The Netherlands

^2^ Laboratory for Optics & Biosciences École Polytechnique, CNRS, INSERM, IP Paris, 91128 Palaiseau Cedex, France

^3^ LaVision BioTec GmbH, a Miltenyi Biotec company, 33617 Bielefeld, Germany

^4^ APE Angewandte Physik & Elektronik GmbH, 13053 Berlin, Germany

^5^ David H. Koch Center for Applied Genitourinary Cancers, The University of Texas MD Anderson Cancer Center, Houston, Texas 77030, USA

^6^ Cancer Genomics Centre, 3584 CG Utrecht, The Netherlands

**Contact details corresponding authors:** Gert-Jan Bakker: email [gert-jan.bakker@radboudumc.nl](about:blank), P +31 (0)24 36 142 96. Peter Friedl: email [peter.friedl@radboudumc.nl](about:blank), P +31 (0)24 36 109 07. Mail address: Dept. of Cell Biology (283) RIMLS, Radboudumc, P.O. Box 9101, 6500 HB Nijmegen, The Netherlands.

**Email addresses co-authors:** Sarah Weischer: [sarah.weischer@radboudumc.nl](about:blank), Júlia Ferrer Ortas: [julia.ferrer-ortas@polytechnique.edu](mailto:julia.ferrer-ortas@polytechnique.edu), Judith Heidelin: [heidelin@lavisionbiotec.de](about:blank), Volker Andresen: [andresen@lavisionbiotec.de](about:blank), Marcus Beutler: [marcus_beutler@ape-berlin.de](about:blank), Emmanuel Beaurepaire: emmanuel.beaurepaire@polytechnique.edu.

**Supplementary File 1a.** Comparison of parameters related to linear, lowIR and highIR excitation modalities.

| Modality | Confocal | lowIR (Ti:Sa/OPO) | 1300 nm highIR | 1650 nm highIR |
| --- | --- | --- | --- | --- |
| Processes | 1-photon, reflection | 2-, 3-photon,  SHG, THG | 3-photon,  SHG, THG | 3-, 4-photon,  SHG, THG |
| Pulse frequency (MHz) | Continuous | 80 | 1 | 1 |
| Pulse length (fs) | - | 140 | 53 | 89 |
| Pulse energy focus (nJ) | - | < 0.4 | < 2 ^a^ | < 7 ^a^ |
| Peak power focus ^b^ | < 1 mW^1,2^ | < 3 kW | 38 kW | 78 kW |
| Power surface (mW) | < 1^2^ | 40-120 | 2.8-33 | 8.7-38 |
| Emission *I(E) ~ …* | *E* | *E^2^, E^3^* | *E^2^, E^3^* | *E^3^, E^4^* |
| Pixel dwell time (µs) | 2 | 2-4 | 10-20 | 10-20 |
| Attenuation of light | very high | moderate | moderate | low |
| Water abs. sample | ~ 0 % | < 3 % | < 6 % | < 26 % |
| Max. depth in tumor (µm) | < 100^2,3^ | 255 | 415 | 395 |

^a)^ Derived from maximum tolerable pulse energy (at the tumor surface, focus 50 µm deep in tissue, Figure 2d, h and i) and effective attenuation lengths for tumor tissue (Figure 3e).

^b)^ Derived as pulse energy in the focus divided by pulse duration.

**Supplementary File 1b.** Order of the excitation processes (*n*) from emission intensity as a function of excitation energy (related to Figure 1).

| Exc. [nm] | Condition | Hoechst | eGFP | TagRFP | mCherry | AF680 | SHG | THG |
| --- | --- | --- | --- | --- | --- | --- | --- | --- |
| 1650 | *In vivo* | 4.2±0.2 | -- | 3.2±0.2 | 3.1±0.4 | 3.0±0.3 | 2.15±0.04 | 3.2±0.1 |
| 1650 | Spheroids | -- | 3.8±0.3 | 3.0±0.2 | 3.0±0.1 | -- | -- | 3.0±0.2 |
| 1650 | Spheroids^a)^ | -- | -- | 3.1±0.1 | 3.0±0.2 | -- | -- | -- |
| 1300 | Spheroids | -- | 2.6±0.4 | 2.5±0.4 |  | -- | 1.85±0.08 | 2.9±0.3 |

^a)^ Measured with the excitation source set to a repetition rate of 0.5 MHz.

**Supplementary File 1c.** Experimental parameters for brain measurements optimized for THG and/or AF680 emission.

| Channel / depth / Wavelength [nm] | Line av. | Port | DM | BP | PMT | Imm. | Seq. |
| --- | --- | --- | --- | --- | --- | --- | --- |
| THG / < 830 µm / 1650 nm | 2 | 2 ch | 525/50 | no | GaAsP | H_2_O | 1 |
| THG / > 830 µm / 1650 nm^a)^ | 6 | 2 ch | 525/50 | no | GaAsP | H_2_O | 2 |
| THG / > 830 µm / 1650 nm | 6 | 2 ch | 880lp | no | GaAsP | D_2_O | 3 |
| AF680 / < 830 µm / 1650 nm | 2 | 4 ch | 900lp | 710/75 | GaAs | H_2_O | 1 |
| AF680 / > 830 µm / 1650 nm | 6 | 2 ch | 880lp | 710/75 | GaAs | H_2_O | 3 |
| THG / < 500 µm / 1270 nm | 3 | 2 ch | 485lp | 417/60 | GaAsP | H_2_O | 4 |
| AF680 / < 500 µm / 1270 nm | 3 | 4 ch | 900lp | 710/75 | Alkali | H_2_O | 4 |
| AF680 / 500-669 µm / 1270 nm | 1 | 4 ch | 900lp | 710/75 | Alkali | H_2_O | 5 |
| AF680 / > 669 µm / 1270 nm | 3 | 4 ch | 900lp | 710/75 | Alkali | H_2_O | 6 |

^a)^ Solely used for generation of the 1650 nm signal attenuation curve.

**Additional References**

1. Pawley, J. B. *Handbook Of Biological Confocal Microscopy*. (Springer, 2006).

2. Centonze, V. E. & White, J. G. Multiphoton excitation provides optical sections from deeper within scattering specimens than confocal imaging. *Biophys. J.* **75**, 2015–2024 (1998).

3. Helmchen, F. & Denk, W. Deep tissue two-photon microscopy. *Nat. Methods* **2**, 932–940 (2005).
