# Supplementary material for: Intravital deep-tumor single-beam 3-photon, 4-photon, and harmonic microscopy
Source: eLife. 2022 Feb 15;11:e63776. doi: 10.7554/eLife.63776 (PMC8849342; doi:10.7554/eLife.63776)
Supplement: Transparent reporting form [file elife-63776-transrepform1.docx]

***eLife’s* transparent reporting form**

We encourage authors to provide detailed information *within their submission* to facilitate the interpretation and replication of experiments. Authors can upload supporting documentation to indicate the use of appropriate reporting guidelines for health-related research (see [EQUATOR Network](http://www.equator-network.org/%20)), life science research (see the [BioSharing Information Resource](https://biosharing.org/)), or the [ARRIVE guidelines](http://www.plosbiology.org/article/info:doi/10.1371/journal.pbio.1000412) for reporting work involving animal research. Where applicable, authors should refer to any relevant reporting standards documents in this form.

If you have any questions, please consult our Journal Policies and/or contact us: [editorial@elifesciences.org](mailto:editorial@elifesciences.org).

**Sample-size estimation**

- You should state whether an appropriate sample size was computed when the study was being designed
- You should state the statistical method of sample size computation and any required assumptions
- If no explicit power analysis was used, you should describe how you decided what sample (replicate) size (number) to use

Please outline where this information can be found within the submission (e.g., sections or figure legends), or explain why this information doesn’t apply to your submission:

Sample size was one, since the image data was used to support the described methodology. The results depended on optimization of technical parameters, rather than describing or depending on events subject to biological variation.

**Replicates**

- You should report how often each experiment was performed
- You should include a definition of biological versus technical replication
- The data obtained should be provided and sufficient information should be provided to indicate the number of independent biological and/or technical replicates
- If you encountered any outliers, you should describe how these were handled
- Criteria for exclusion/inclusion of data should be clearly stated
- High-throughput sequence data should be uploaded before submission, with a private link for reviewers provided (these are available from both GEO and ArrayExpress)

Please outline where this information can be found within the submission (e.g., sections or figure legends), or explain why this information doesn’t apply to your submission:

The study of the threshold for nonlinear phototoxicity (phototoxicity limited to the focal plane) was updated and includes technical replicates and statistical analysis (Revised Figure 2d).

Imaging measurements were not replicated, since the image data was used to support the described methodology. The results depended on optimization of technical parameters were not replicated.

**Statistical reporting**

- Statistical analysis methods should be described and justified
- Raw data should be presented in figures whenever informative to do so (typically when N per group is less than 10)
- For each experiment, you should identify the statistical tests used, exact values of N, definitions of center, methods of multiple test correction, and dispersion and precision measures (e.g., mean, median, SD, SEM, confidence intervals; and, for the major substantive results, a measure of effect size (e.g., Pearson's r, Cohen's d)
- Report exact p-values wherever possible alongside the summary statistics and 95% confidence intervals. These should be reported for all key questions and not only when the p-value is less than 0.05.

Please outline where this information can be found within the submission (e.g., sections or figure legends), or explain why this information doesn’t apply to your submission:

Figure 1d and Supplementary Figure 4: the fitting algorithm is described in Materials and Methods section. Figure 2c: the number of cells used per averaged intensity time trace is mentioned in the figure. Figure 2d: the number of measurements and cells per condition, the statistical test and p-value are mentioned in the figure caption. Figure 2h, i: the number of measured cells per curve is mentioned in the figure. Figure 3d: the number of features analyzed per datapoint are given in figure caption, exact values can be found in the source data file. Supplementary Figure 5b: the test criterium for the moment of onset of Sytox- or GCaMP-positivity within single cell intensity time traces is described in the figure caption. Supplementary Figure 5f: the number of measurements and cells per condition, the statistical test and p-value are mentioned in the figure caption.

(For large datasets, or papers with a very large number of statistical tests, you may upload a single table file with tests, Ns, etc., with reference to sections in the manuscript.)

**Group allocation**

- Indicate how samples were allocated into experimental groups (in the case of clinical studies, please specify allocation to treatment method); if randomization was used, please also state if restricted randomization was applied
- Indicate if masking was used during group allocation, data collection and/or data analysis

Please outline where this information can be found within the submission (e.g., sections or figure legends), or explain why this information doesn’t apply to your submission:

For determination of the nonlinear phototoxicity threshold, measurements with excitation energies below 12 nJ showed a similar, low response. These measurements (2x5.0 and 2x8.4 nJ) were pooled to obtain a large enough group size for statistical tests (Results and Discussion section about Phototoxicity).

**Additional data files (“source data”)**

- We encourage you to upload relevant additional data files, such as numerical data that are represented as a graph in a figure, or as a summary table
- Where provided, these should be in the most useful format, and they can be uploaded as “Source data” files linked to a main figure or table
- Include model definition files including the full list of parameters used
- Include code used for data analysis (e.g., R, MatLab)
- Avoid stating that data files are “available upon request”

Please indicate the figures or tables for which source data files have been provided:

Source data files, including raw image data, have been supplied for all images, videos and for the Supplementary File 1. The numerical data, data fitting, statistical calculations and rendering behind presented data plots are available on the journal’s website (…-source data 1). The microscopy image data, intermediate and analysis results that support the findings of this study (…-source data 2) are available in Figshare (<https://figshare.com/projects/Intravital_Deep-Tumor_Single-Beam_3-Photon_4-Photon_and_Harmonic_Microscopy/89273> and direct links in figure captions). Analysis scripts have been supplied and have been referenced in the Materials and Methods section (…-Source Code 1, 2, …).
